# A simple strategy for managing many recessive disorders in a dairy cattle breeding program

**DOI:** 10.1186/s12711-015-0174-9

**Published:** 2015-11-30

**Authors:** John B. Cole

**Affiliations:** Animal Genomics and Improvement Laboratory, Agricultural Research Service, United States Department of Agriculture, Beltsville, MD USA

## Abstract

**Background:**

High-density single nucleotide polymorphism genotypes have recently been used to identify a number of novel recessive mutations that adversely affect fertility in dairy cattle, as well as to track other conditions such as red coat color and polled. Most current methods for mate allocation fail to consider this information, and it will become increasingly difficult to manage matings as the number of recessive mutations to be accounted for increases.

**Methods:**

A modified version of a mating strategy that constrains inbreeding based on genomics (the Pryce method) was developed that also accounts for the economic effects of Mendelian disorders on overall economic merit (modified Pryce method) and compared with random mating, truncation selection, and the Pryce scheme. Several scenarios were considered, including scenarios with six hypothetical recessive alleles and 12 recessive alleles that are currently segregating in the US Holstein population.

**Results:**

The Pryce method and the modified Pryce method showed similar ability to reduce frequencies of recessive alleles, particularly for loci with frequencies greater than 0.30. The modified Pryce method outperformed the Pryce method for low-frequency alleles with small economic value. Cumulative genetic gain for the selection objective was slightly greater when using the Pryce method, but rates of inbreeding were similar across methods.

**Conclusions:**

The proposed method reduces allele frequencies faster than other methods, and also can be used to maintain or increase the frequency of desirable recessives. It can be easily implemented in software for mate allocation, and the code used in this study is freely available as a reference implementation.

## Background

Recessive disorders have been identified in livestock populations since modern animal breeding programs began, and hundreds of these disorders have now been catalogued [1]. In the past, test matings were used to identify carriers of recessive disorders [2], but most recessive mutations were identified after the carrier bull had sired many daughters and had sons used for artificial insemination (AI) (e.g., bovine leukocyte adhesion deficiency [3], complex vertebral malformation [4], and deficiency of uridine monophosphate synthase [5]). Novel recessive mutations can also quickly spread through a population by using popular bulls before routine screening is possible because such defects are not directly observable, e.g., Jersey haplotype 1 [6].

Several authors have proposed methods for including information on quantitative trait loci (QTL) in breeding programs. Most approaches focus on the calculation of the additive genetic value of a QTL, which is then combined with other information using a selection index approach [7–10]. Shepherd and Kinghorn [11] described how QTL information can be included in a look-ahead mate selection scheme, and they have suggested that this could be incorporated into a comprehensive mating service, such as Total Genetic Resource Management™ ([12]; http://www.xprime.com.au/products/tgrm/index.html), once efficient algorithms have been developed. Li et al. [13, 14] reported that the use of QTL genotypes provides more benefit when used in mate selection rather than in index selection for a variety of inheritance modes under several breeding structures. Recently, Van Eenennaam and Kinghorn [15] extended the MateSel program [16] to permit selection against the total number of lethal alleles and recessive lethal genotypes.

Genomic tools have enabled the detection of many new recessive alleles that have deleterious effects on fertility [17], many of which have effects early in gestation and could not previously be distinguished from failed breedings. Since the number of identified recessive mutations continues to increase, new tools are needed to consider such information when making mating decisions. However, many mate allocation tools do not consider carrier status when bulls and cows are paired, and few make use of the DNA marker or haplotype information that is increasingly available for bulls and cows. If only a few recessive alleles segregate in a population, it is easy to monitor individuals to avoid carrier-to-carrier matings, but this becomes considerably more difficult, or even impossible, as their number increases.

Pryce et al. [18] recently proposed a simple method for controlling the rate of genomic inbreeding increase by penalizing parent averages (PA) for matings that produce inbred offspring. After PA are adjusted, the bull that produces the highest PA when mated to a cow is selected in a sequential manner, while the number of matings permitted for each bull is constrained to prevent one bull from being mated to all cows. This method is straightforward to program and effectively constrains genomic inbreeding at reasonable levels. The objectives of this research were to extend the Pryce method to include information on recessive alleles and to examine its use to simultaneously account for a large number of Mendelian disorders when allocating mates in dairy cattle breeding schemes. Managing genetic defects is a trade-off between avoiding matings between carriers in the short-term and eliminating defects in the long run, so a simulation model was used to examine long-term changes in the population.

## Methods

Computer simulation was used to compare four systems of mating applied to scenarios including hypothetical recessive alleles, as well as 12 recessive alleles that are currently segregating in the US Holstein population.

### Base population

Base population cows had true breeding values (TBV) for lifetime net merit (NM$) that were randomly sampled from a normal distribution with a mean of $0 and a standard deviation of $200, which is similar to the genetic standard deviation (SD) of lifetime net merit [19]. Bull TBV were sampled from a normal distribution with a mean of $300 (+1.5 genetic SD of NM$) and a SD of $200. An animal’s carrier status for each recessive allele was determined by randomly sampling sire and dam alleles using the minor allele frequencies (MAF) in Table 1. Recessive alleles were assumed to be independent of one another, i.e., assuming each locus was located on a different chromosome. A sex ratio of 0.5 was used and base population animals were assigned a birth year from −9 to 0 (bulls) or −4 to 0 (cows) by sampling from a uniform distribution.

**Table 1:** Properties of the recessive loci included in each scenario simulated

Group	Scenario^a^	N^b^	Recessive loci			
			Frequency	Value ($)^c^	Name	Lethal
Holstein	All recessive loci	12	0.0276	150	Brachyspina	Yes
			0.0192	40	HH1	Yes
			0.0166	40	HH2	Yes
			0.0295	40	HH3	Yes
			0.0037	40	HH4	Yes
			0.0222	40	HH5	Yes
			0.0025	150	BLAD	Yes
			0.0137	70	CVM	Yes
			0.0001	40	DUMPS	Yes
			0.0007	150	Mulefoot	Yes
			0.9929	40	Horned	No
			0.0542	−20	Red coat color	No
	All recessive loci, high cost	12	0.0276	450	Brachyspina	Yes
			0.0192	120	HH1	Yes
			0.0166	120	HH2	Yes
			0.0295	120	HH3	Yes
			0.0037	120	HH4	Yes
			0.0222	120	HH5	Yes
			0.0025	450	BLAD	Yes
			0.0137	210	CVM	Yes
			0.0001	120	DUMPS	Yes
			0.0007	450	Mulefoot	Yes
			0.9929	120	Horned	No
			0.0542	−60	Red coat color	No
Hypothetical	High frequency, low value	1	0.90	20	High, low	Yes
	High frequency, high value	1	0.90	200	High, high	Yes
	Medium frequency, low value	1	0.50	20	Medium, low	Yes
	Medium frequency, high value	1	0.50	200	Medium, high	Yes
	Low frequency, low value	1	0.01	20	Low, low	Yes
	Low frequency, high value	1	0.01	200	Low, high	Yes
	All recessive loci	6	As above			
Horned	Horned locus, market value	1	0.9929	40	Horned	No
	Horned locus, high value	1	0.9929	400	Horned	No

^a^Specific scenario simulated for each trait or group of traits

^b^Number of recessive loci in the scenario

^c^Positive values are undesirable and negative values are desirable

The base population in each scenario included 350 bulls and 35,000 cows distributed over 200 herds, and the population was permitted to grow to a maximum of 500 bulls and 100,000 cows over the 20 generations simulated. Bulls were permitted a maximum of 5000 matings per year, and in the truncation selection scheme described later in this section only the top 10 % of bulls based on TBV were retained for use as mates.

### Descendants

The TBV for new calves were created by taking the PA and adding a Mendelian sampling term:

$${\text{TBV}}_{\text{calf}} = \, 0. 5\left( {{\text{TBV}}_{\text{sire}} + {\text{ TBV}}_{\text{dam}} } \right) \, + {\text{ MS}},$$

where TBV_calf_, TBV_sire_, and TBV_dam_ are the TBV of the calf, its sire, and its dam, respectively. The Mendelian sampling term, MS, was drawn from a normal distribution with a mean of 0 and a variance of $$\frac{1}{2}\left[ {1 - \frac{1}{2}\left( {f_{S} + f_{D} } \right)} \right]\sigma_{a}^{2}$$, where f_S_ and f_D_ are the coefficients of inbreeding of the sire and dam, respectively, and $$\sigma_{a}^{2}$$ is the additive genetic variance of NM$ ($40,000). Sex was assigned randomly with a 50:50 sex ratio. Calves were born in the same herd as their dams, and cows did not move between herds. For each recessive locus, one allele was sampled at random from each parent and used to construct the progeny genotype. Both lethal [e.g., deficiency in uridine monophosphate synthase (DUMPS)] and non-lethal (e.g., red coat color) recessive alleles were included in the simulations. Recessive genotypes were simulated without error, and it was only necessary to simulate genotypes for recessive alleles because pedigrees were assumed to be free of errors. If the recessive locus was lethal, an affected (aa) calf was created and marked as dead. Allele frequencies were updated each generation by counting alleles.

Each step in the simulation represented 1 year of calendar time. New animals were born at the beginning of each year, affected calves died, and surviving animals were culled for age, to maintain population size, and at random (when enabled) at the end of each round (year) of simulation. Generations overlapped and bulls and cows could have offspring in multiple years. Bulls were culled first for age, with a maximum age of 10 years, and then on TBV (lowest-ranking animals were culled first) to maintain a maximum population size. Cows were first culled for age, with a maximum age of 5 years. After age-related culling, animals were culled involuntarily. Finally, cows were culled at random to maintain population size, if necessary. Animals were not culled based on carrier status, and cows were not culled because of abortions or stillbirths.

### Mating schemes

Four systems of mating, referred to hereafter as schemes, were simulated: random mating, truncation selection, the scheme proposed by Pryce et al. [18], and a modified version of the Pryce scheme that accounts for recessive alleles. In the random mating scheme, all bulls were mated randomly to cows, while limiting the maximum number of matings permitted for each bull (5000). In the truncation selection scheme, the top 10 % of bulls, based on TBV, were randomly mated to the cow population with no limit on the number of matings permitted to each bull.

In the Pryce scheme, matings were assigned as follows. For each herd, 20 % of the bulls were randomly selected from the list of live bulls, and the top 50 bulls from that group were selected as herd sires based on TBV. As a result, the bulls for each herd were selected from a slightly different portfolio of animals. This strategy is similar to that used by Pryce et al. [18] for cows and bulls. A matrix of PA, **B**_**0**_, was then constructed with rows corresponding to bulls and columns corresponding to cows. The elements of **B**_**0**_ were computed as:

$${\text{B}}_{\text{ij}} = \, 0. 5\left( {{\text{TBV}}_{\text{i}} + {\text{ TBV}}_{\text{j}} } \right) \, - \, \lambda {\text{F}}_{\text{ij}} ,$$

where TBV_i_ is the TBV for NM$ of bull *i*, TBV_j_ is the TBV for NM$ of cow *j*, λ is the inbreeding depression ($) associated with a 1 % increase in inbreeding, and F_ij_ is the pedigree coefficient of inbreeding of the calf resulting from mating bull *i* to cow *j*. The regression coefficient of NM$ on inbreeding (λ) was computed as the weighted average of the effects of inbreeding [20] on the traits in the index, with weights corresponding to those assigned to each trait in NM$. A value for λ of $25 was used, which is similar to the $23.11 calculated by Weigel and Lin [21], but higher than the $12 reported by Smith et al. [22] and the AUS$5 value used by Pryce et al. [18]. In the fourth scheme, the elements of **B**_**0**_ were adjusted to account for the recessive alleles carried by the parents as:

$$B_{ij}^{'} = B_{ij} - \mathop \sum \limits_{r = 1}^{{n_{r} }} P\left( {aa} \right)_{r} \times v_{r} ,$$

where n_r_ is the number of recessive alleles in a scenario, P(aa)_r_ is the probability of producing an affected calf for recessive locus *r*, and v_r_ is the economic value of *r*. P(aa) will be equal to either 0.25, for a mating of two carriers, 0.5, for a mating of an affected animal with a carrier, or 1, for a mating of two affected animals. The recessive loci used in each scenario are described in Table 1, which includes the MAF and the economic value assigned to each. For each recessive locus, F_ij_ is correlated with P(aa), which will result in some double-counting of the economic impact of each locus, and this may produce suboptimal rates of genetic gain. The relationship of these two quantities is discussed in detail in the “Results and discussion” section.

Once matrix **B** (or **B**′, depending on the scenario) is constructed, a matrix of matings, **M**, is used to allocate bulls to cows. An element, M_ij_, is set to 1 if the corresponding value of B_ij_ is the greatest value in column *j* (that bull produces the largest PA of any bull available for mating to cow *j*), and all the other elements of column *j* are set to 0. If the sum of the elements of row *i* is less than the maximum number of permitted matings for that bull, then the mating is allocated. Otherwise, the bull with the next-highest value of B_ij_ in the column is selected, and so on, until each column has one and only one element equal to 1. This approach overestimates genetic progress because it assumes equal selection accuracy among male and female breeding values, whereas, in practice, selection accuracy is lower in females, but it allows a reasonable comparison of the Pryce and modified Pryce algorithms. All animals were assumed to be genotyped so that recessive status is known.

### Recessive scenarios

Several scenarios were used to characterize the performance of the proposed method. The scenarios differed in the number and economic value of recessive loci considered.

#### Economic values

Each recessive locus was assigned an economic value based on the occurrence of embryonic or foetal loss during pregnancy (for lethal alleles), or on literature values for non-lethal conditions such as red coat color and horned status. Since Holstein haplotypes 1 through 5 (HH1 to HH5) and DUMPS occur early in pregnancy, they were assigned a value of $40 based on reproductive costs (e.g., cost of semen, value of days open) included in the 2014 revision of the NM$ index [19]. Brachyspina and mulefoot result in stillbirths or calves that do not survive until adulthood and they were assigned relatively high costs of $150, although actual losses could be higher. Complex vertebral malformation (CVM) results in late-term abortions, so an intermediate value between that of the Holstein fertility haplotypes and brachyspina/mulefoot was used. The high-cost scenario used three times the cost of the base scenario to assess the sensitivity of results to economic values. For the hypothetical recessive loci, economic values of either 0.10 ($20) or 1 ($200) genetic SD of NM$ were used. Non-lethal recessive loci had an economic value of either 0 (in the case of recessive alleles with no effect on lifetime profitability) or had a negative value (which increases the PA for economically desirable recessive alleles such as polled).

#### Holstein recessive loci

Twelve recessive loci that currently segregate in the US Holstein population were used to determine how the modified Pryce method performed in a commercial livestock population: bovine leukocyte adhesion deficiency (BLAD), brachyspina, CVM, DUMPS, HH1–HH5, horned, mulefoot, and red coat color. Two scenarios that included the 12 Holstein recessive loci but differed in the economic value assigned to each locus, were used to determine the sensitivity of matings to different prices. In the normal scenario, prices were assigned based on the effect of the recessive locus and the timing of occurrence, as described above. For the high-cost scenario, the prices used for the normal scenario were multiplied by three. Frequencies for the 12 recessive alleles were taken from [23].

#### Hypothetical recessive loci

The effect of initial allele frequency on response to selection under each strategy was examined using six scenarios. Each scenario included a single recessive locus but differed in the initial frequency of the recessive allele: low (0.01), medium (0.50), or high (0.90); and in the economic value of the recessive: low ($20) or high ($200). In addition, a seventh scenario that included all hypothetical loci was examined.

#### Horned and other high-frequency non-lethal recessive conditions

Not all recessive alleles in a livestock population are lethal, one example being the horned locus in cattle. Because the horned condition in cattle is due to the action of a recessive allele [24] with a very high frequency, it was included in the simulation in place of polled, with an allele frequency of 1 − 0.0071 = 0.9929. Based partly on the work of Widmar et al. [25], who calculated an average expected cost of dehorning of $22.52, a value of $40 was assumed for the horned genotype to also account for breeders’ preferences and premium marketing opportunities. Recall that a positive value reduces the PA in the modified Pryce method, which in this case results in a lower frequency of horned individuals.

#### Many recessive loci

Scenarios with 100 and 1000 recessive loci were run in order to examine the relationship of inbreeding with the probability that an embryo will be affected by one or more recessive conditions [the sum of the P(aa) terms within an individual] using the modified Pryce method. Initial MAF were sampled from a uniform distribution in the interval [0.01, 0.10] and the economic values were sampled from a uniform distribution in the interval [−$10, −$50]. Correlations of embryo inbreeding with the sum of P(aa) were calculated, and separate regressions of the probability that an embryo will be affected by one or more recessive conditions on inbreeding were computed for the assigned matings and for all other possible matings.

### Analysis

Results were averaged over each of 10 replicates for each scenario. Observed changes in allele frequency were compared against expectations, where the expected allele frequency at each generation for lethal alleles was calculated using an equation derived from Van Doormaal and Kistemaker [26]:

$$p_{t} = \frac{{p_{t - 1}^{2} + p_{t - 1} q_{t - 1} }}{{2p_{t - 1}^{2} + p_{t - 1} q_{t - 1} }},$$

$$q_{t} = \frac{{p_{t - 1} q_{t - 1} }}{{2p_{t - 1}^{2} + p_{t - 1} q_{t - 1} }},$$

where *p*_*t*_ is the frequency of the major allele at time *t*, *q*_*t*_ is the frequency of the minor allele at time *t*, and *t* is the time in years (ranging from 1 to 20). The MAF at time 0 was the value used in each scenario for each recessive locus and the major allele frequency was calculated as 1 minus MAF. Expected frequencies for non-lethal alleles were calculated based on Hardy–Weinberg proportions [27] as:

$$\begin{array}{*{20}c} {p_{t} = p_{t - 1}^{2} + p_{t - 1} q_{t - 1} } \\ {q_{t} = q_{t - 1}^{2} + p_{t - 1} q_{t - 1} } \\ \end{array} .$$

For each recessive locus in each scenario, actual and expected allele frequencies were regressed on generation with the Python module Statsmodels version 0.5.0 ([28]; http://statsmodels.sourceforge.net/) using the model:

$$y_{t} = b_{0} + b_{1} g_{t} + e_{t}$$

where *y*_*t*_ is the frequency of a recessive locus at time *t*, *b*_*0*_ is the intercept term, *b*_*1*_ is the slope, *g*_*t*_ is the generation number at time *t*, and *e*_*t*_ is the random residual error. Two different comparisons were made using the slopes from these regressions: actual allele frequencies were compared to expected allele frequencies within methods to determine if allele frequencies were changing faster (or slower) than expectations, and actual allele frequencies were compared across methods to determine if allele frequencies were changing more quickly based on the method used. Slopes were extracted from regression results and a two-sample *t* test assuming unequal variances was used to compare the coefficients against each other. A Bonferroni adjustment was used to correct for multiple comparisons.

### Computing environment

All simulations were performed on a Pogo Linux Atlas 1205 (Pogo Linux, Inc., Redmond, WA) computer with an 8-core AMD Opteron 6328 processor with a clock speed of 3.2 GHz, 64 GB of DDR3 1600 MHz RAM, and 64-bit CentOS Linux EL6 (Red Hat, Inc., Raleigh, NC), or a Thinkmate RAX QS6-4210 (Thinkmate, Inc., Waltham, MA) workstation with four 12-core AMD Opteron 6344 processors with a clock speed of 2.6 GHz, 256 GB of DDR3 1600 MHz RAM, and CentOS Linux EL7. Data analysis and visualization were performed on a MacBook Pro with two Intel Core i7 processors running at 2.9 GHz, 8 GB of DDR3 1600 MHz RAM, and Mac OS X 10.7.5 (Apple Inc., Cupertino, CA).

Computation time for the random mating scheme averaged 193 min per replicate for a one recessive locus scenario (high frequency, high cost) and 215 min for a 12 recessive loci scenario (Holstein recessive loci). Considerably less time was required for the truncation selection scheme, averaging 34 min for the one recessive locus scenario and 38 min in the Holstein scenario. The time needed for the Pryce and modified Pryce schemes averaged 206 and 232 min for the one recessive locus scenario, and 203 and 279 min for the Holstein scenario. These two schemes required the allocation of large arrays and the creation of large output files that were not needed for the random mating or truncation selection schemes. If matings are done within herd, the memory used for one herd can be reused for the next herd to keep memory requirements low. The time required for processing one generation rather than 20 should be very reasonable (~1/20 of the times presented above).

## Results and discussion

### Holstein recessive loci

#### Normal cost scenario

**Fig. 1:**
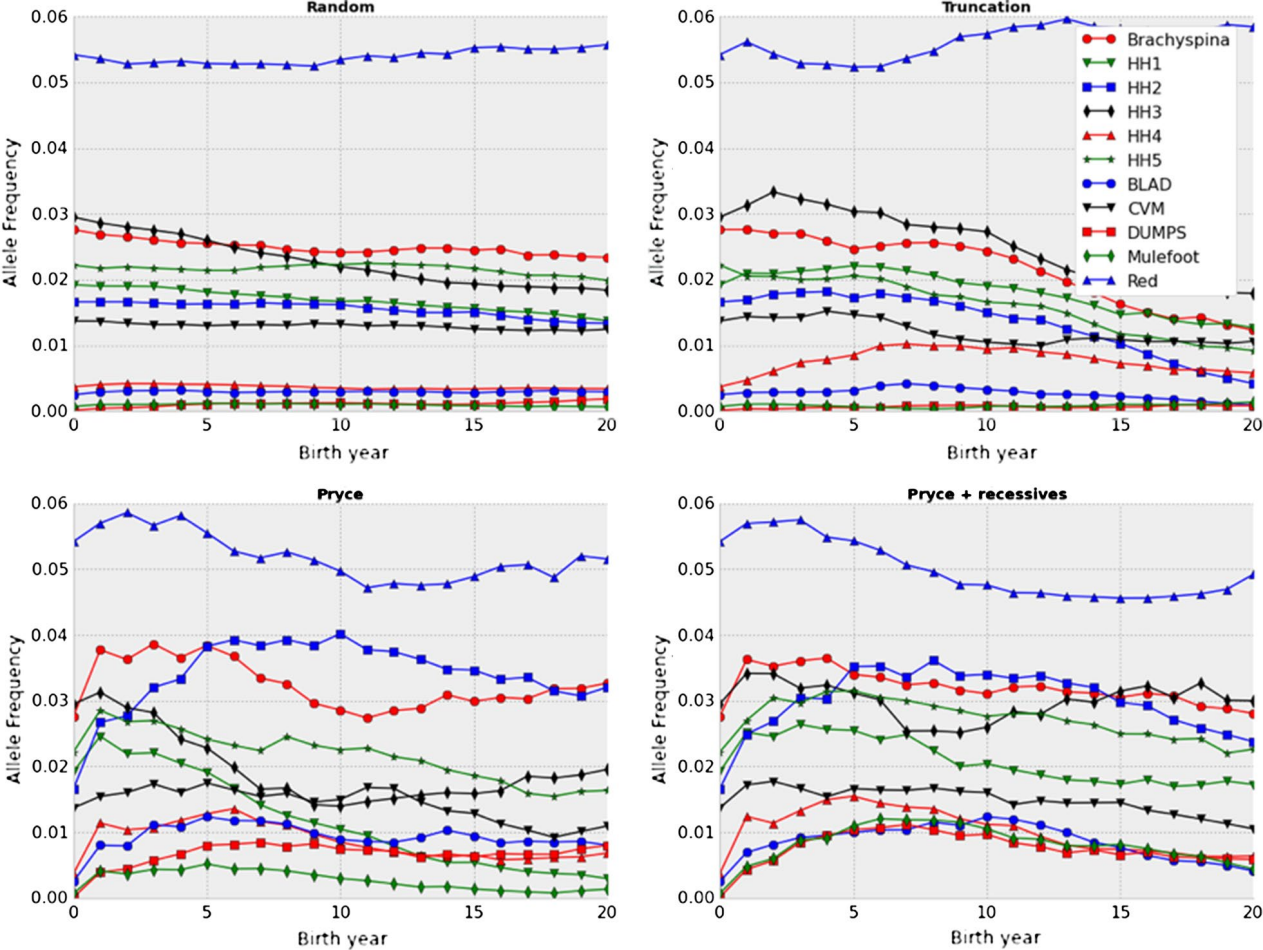
Observed frequencies of Holstein recessive alleles. Observed changes in minor allele frequencies for BLAD, brachyspina, CVM, DUMPS, HH1–HH5, mulefoot, and red coat color over 20 years under random selection, truncation selection, the Pryce method for controlling genomic inbreeding, and the modified Pryce method that accounts for recessive loci

**Fig. 2:**
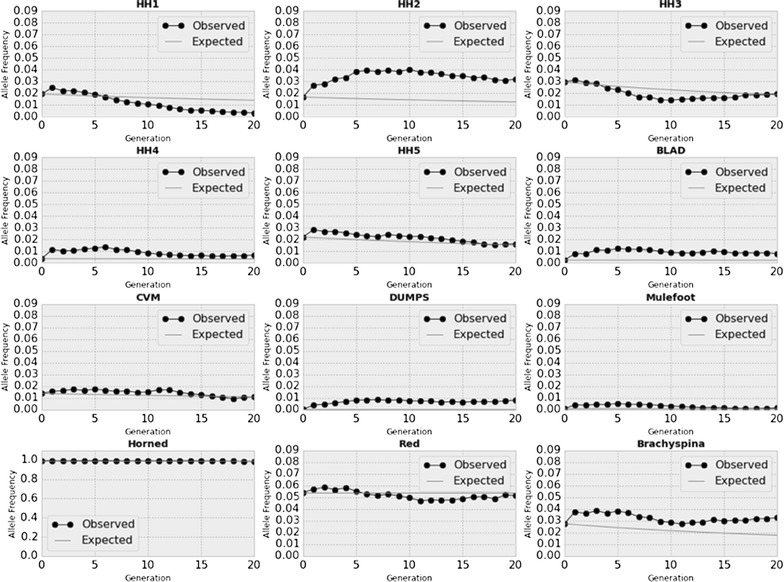
Observed versus expected allele frequencies of Holstein recessive alleles under the Pryce scenario. Observed versus expected changes in minor allele frequencies for BLAD, brachyspina, CVM, DUMPS, HH1–HH5, horned, mulefoot, and red coat color over 20 years using the Pryce method for controlling genomic inbreeding. Note that the horned subplot is scaled differently on the y axis than the other subplots because of the horned allele frequency

Changes in allele frequencies for 11 of the 12 recessive loci resulting from the four mating schemes are in Fig. 1. Frequencies are not shown for horned locus because the allele frequency remained above 99 % for all four schemes. The frequency of the 10 lethal alleles generally decreased over time in all scenarios. The frequencies of the HH1 and HH3 alleles decreased significantly faster (P < 0.05 after a Bonferroni correction) with the Pryce scheme than with the modified Pryce scheme. The rate of change in allele frequencies was similar for the Pryce (Fig. 2) and modified Pryce (data not shown) schemes. An advantage of the modified Pryce scheme is that it maintains the frequency of desirable recessive alleles, such as red coat color, in the population. With the Pryce scheme, the frequency of the red coat color allele decreased over time because with that scheme, there is no mechanism to balance undesirable economic effects of inbreeding against the desirable economic value of some recessive individuals. In the modified Pryce scheme, the positive economic value of red coat color offset the inbreeding penalty and it maintained a relatively constant gene frequency over time. Avoidance of genomic inbreeding limits homozygosity, but eventually the population should become homozygous for the favorable allele.

Average TBV over time for the total merit index under selection were similar for the Pryce and modified Pryce schemes. The difference in average TBV of cows in year 20 was $21, i.e., $2966 versus $2987 for the Pryce and modified Pryce schemes, respectively. Bulls in generation 20 differed by only $10 on average ($3737 versus $3747). These differences were relatively small compared to the genetic gain in the population, which averaged approximately $148 per year for cows and $186 per year for bulls.

Average coefficients of inbreeding per birth year were very similar for cows and bulls and increased by approximately 0.35 % per year in both populations. The same general pattern was observed across all scenarios and mate allocation schemes (data not shown).

#### High cost scenario

**Fig. 3:**
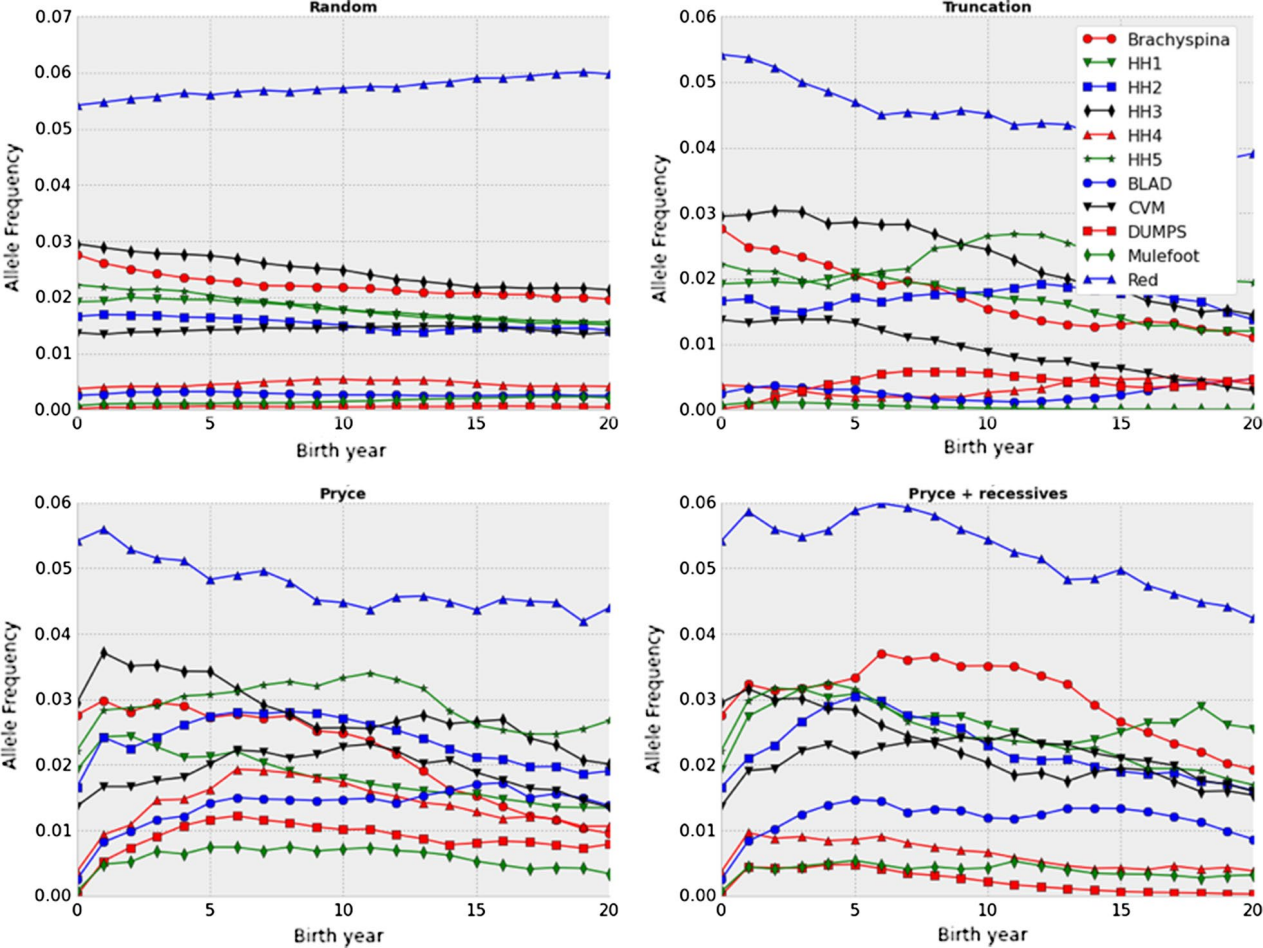
Observed allele frequencies for Holstein recessive loci with high economic values. Observed changes in minor allele frequencies for BLAD, brachyspina, CVM, DUMPS, HH1–HH5, mulefoot, and red coat color over 20 years under random selection, truncation selection, the Pryce method for controlling genomic inbreeding, and the modified Pryce method that accounts for recessive alleles

When the economic value of each recessive locus was increased by a factor of 3 over that in the base Holstein scheme, results were similar to those of the base Holstein scenario (Fig. 3). Frequencies of the HH5 and horned alleles decreased faster with the modified Pryce scenario than with the Pryce scenario (P < 0.05), while that of the HH1 allele decreased faster with the Pryce scenario.

### Hypothetical recessive loci

#### High frequency, lethal recessive alleles

**Fig. 4:**
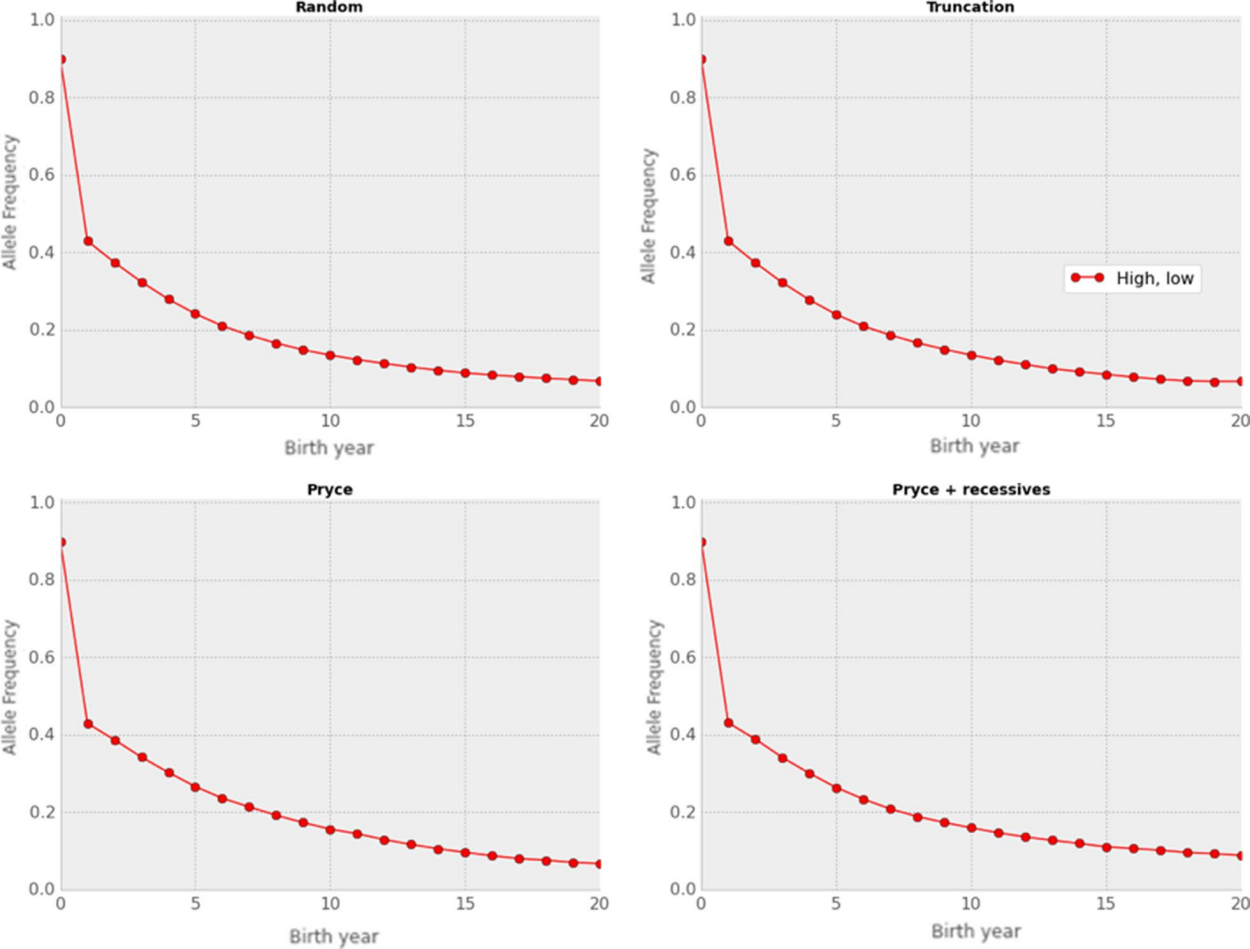
Observed frequencies for a hypothetical recessive allele with a high frequency and a low economic value. Observed changes in minor allele frequency for a hypothetical recessive allele with a starting frequency of 0.90 and an economic value of $20 over 20 years under random selection, truncation selection, the Pryce method for controlling genomic inbreeding, and the modified Pryce method that accounts for recessives

The rate of change in allele frequencies was similar for both the low ($20; Fig. 4) and high ($200; data not shown) value scenarios. This suggests that at high minor allele frequency the change from generation to generation is driven principally by genotype frequencies, not by the economic value. The fit of the observed to expected allele frequency changes was very good for both scenarios (data not shown).

#### Medium frequency, lethal recessive alleles

Results for a minor allele with an initial frequency of 0.50 and an economic value of either $20 or $200 were very similar to those for the high frequency scenario. Initial allele frequencies again had a much larger effect on final frequencies than economic values, and a different mate allocation strategy will be needed to decrease the allele frequency more quickly in these cases.

#### Low frequency, lethal recessive alleles

The low-frequency scenario is representative of the recessive conditions that are most commonly seen in livestock populations [23], i.e., harmful alleles have low frequencies (<0.05). Both the Pryce and modified Pryce methods were successful at decreasing the allele frequency over time, when the value of the recessive locus was high, and it was faster than expected. However, the modified Pryce method appeared to be more effective at lowering the allele frequency than random mating, truncation selection, or the Pryce scheme when the economic value of the recessive locus was low.

#### Six hypothetical, lethal recessive loci

All four systems of mate allocation produced similar changes in allele frequencies over time. The Pryce method and the modified Pryce method did produce slightly lower frequencies for some of the recessive alleles that had high or medium initial frequencies, but there was no apparent pattern based on the economic value of each locus. Observed allele frequencies showed much better fits to the predicted values than in the scenarios based on the actual Holstein recessive loci, but that is as expected when alleles have initial frequencies greater than 0.20.

There was no apparent difference in the rate of change in allele frequencies over time although there was a tenfold difference in economic values between scenarios with high ($200) and low ($20) values for the recessive loci. When MAF was high, many of the potential mate pairs in the population will have reduced PA, but the loci with large economic values will be penalized more than those with low values, which should result in fewer carrier-to-carrier matings. Increasing the economic value of recessive loci is not sufficient to increase the rate at which undesirable alleles are eliminated from the population.

### Horned and other high-frequency non-lethal recessive loci

The horned allele is present at a frequency greater than 99 % in the US Holstein population and there is increasing interest in reducing its frequency to improve animal welfare. A scenario that only included the horned recessive locus was simulated to determine if the modified Pryce scheme is an effective tool to reduce the frequency of horned animals in the population, i.e., increasing the frequency of polled. A $40 value for the horned locus was not effective in reducing MAF, probably because the frequency of the polled allele is so low that carriers were unlikely to be one of the top-ranked bulls based on TBV. The limitation of 5000 matings per generation also limited the effect of a high TBV polled bull on the population. Increasing the value of the horned locus from $40 to $400 was also unsuccessful in changing the allele frequency. These results are consistent with those of the 12 recessive loci scenarios described above, in which there was no change in the frequency of the horned allele. Thus, a more sophisticated approach for selecting mate pairs that will either produce polled offspring or carriers, such as a scheme described by Li et al. [13, 14] or Spurlock et al. [29], or the use of tools for non-meiotic allele introgression [30], are necessary to effectively increase the frequency of polled (decrease the frequency of horned) cows in the national dairy herd.

### Mating schemes

As expected, genetic trend was negligible under the random mating scheme, except in scenarios in which lethal loci had an initial MAF greater than 20 %. Overall, the genetic trend from the truncation selection scheme was similar to those from the Pryce and modified Pryce schemes for lethal recessive loci, and from the random mating scheme for non-lethal recessive loci. This is reasonable because the frequency of individuals with a lethal condition is expected to decrease over time even if no additional selection pressure is imposed, and the threshold that retains the top 10 % of bulls for breeding ensures that genetic trend is positive. The truncation selection scheme loosely resembles current mating strategies used on large commercial dairies in North America.

**Fig. 5:**
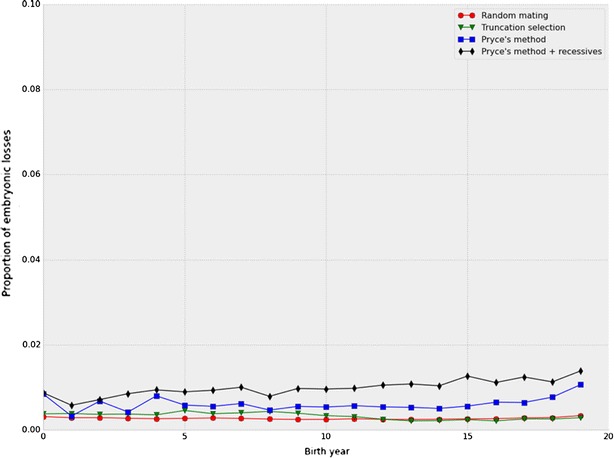
Embryonic deaths by birth year. Proportion of embryos in each birth year that died due to the effects of recessive genotypes

More affected calves were observed in the Pryce and modified Pryce schemes than in the random mating and truncation selection schemes. Figure 5 shows the proportion of simulated calves that were culled due to having homozygous recessive genotypes averaged over replicates for the Holstein scenario; results were similar for the high value and high frequency scenario and for the low value and low frequency scenario (data not shown). This is as expected because a bull can have a greater genetic superiority over its contemporaries than the value of the recessive alleles it may carry. Selection for a smaller total number of recessive alleles carried by an animal, rather than a lower frequency of homozygous recessive genotypes, could result in fewer embryonic losses [15]. Thus, there is a conflict between the goal of eliminating recessive alleles from the population, which involves fixing associated haplotypes in a homozygous state, and the goal of minimizing inbreeding, which seeks to avoid such increases in homozygous individuals.

### Relationships of inbreeding with recessive load

**Fig. 6:**
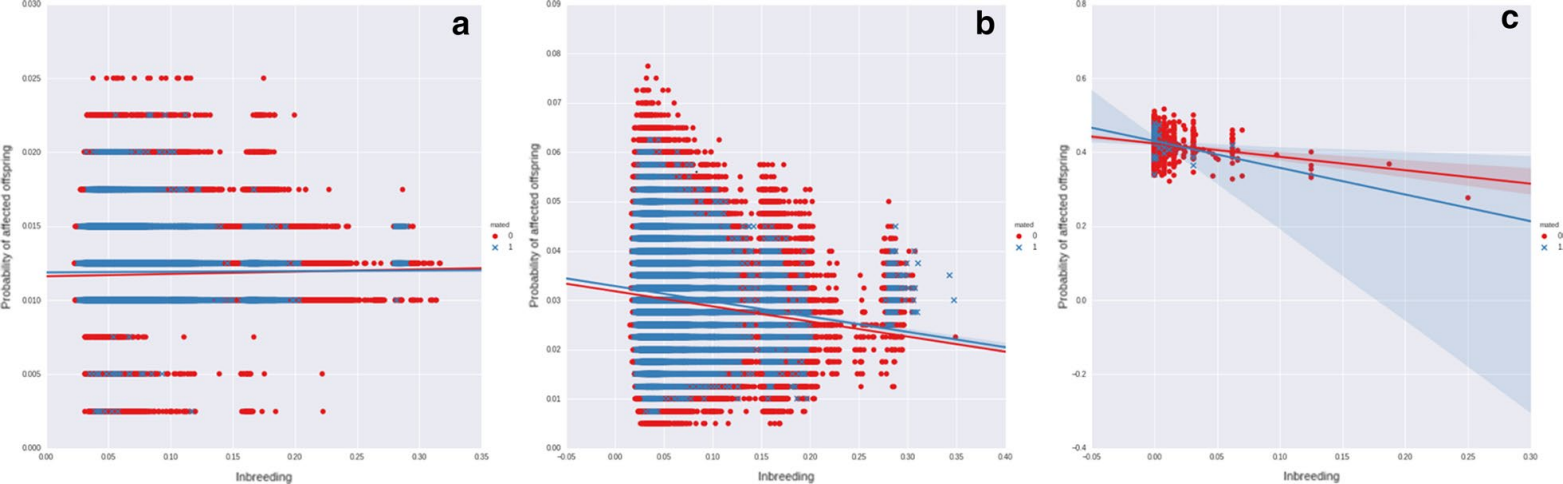
Embryo inbreeding and probability of carrying recessive alleles. Relationships between embryo inbreeding and the probability that the embryo will be affected by one or more recessive conditions for **a** Holstein recessive alleles, **b** 100 simulated recessive alleles, and **c** 1000 simulated recessive alleles. Possible matings that were not made (*red dots*) are distinguished from those that were made (*blue crosses*)

The relationship between inbreeding and the number of recessive alleles carried by parents was examined by computing the correlation of F_ij_ with the sum of P(aa) for each possible mating in each generation (ΣP(aa)) for scenarios with 12 (the Holstein scenario discussed above), 100, or 1000 recessive loci. Contrary to expectations, the correlation of F_ij_ with ΣP(aa) was near 0 in the Holstein scenario, and negative in the 100 and 1000 recessive loci scenarios. The correlation was stronger for matings that were made than for matings that were not made, which suggests that the modified Pryce method was successful in identifying matings that reduced the accumulation of recessive alleles. Figure 6a–c shows the regressions of the probability that an embryo will be affected by one or more recessive conditions on inbreeding for the matings that were evaluated in birth year 20 of replicate 1 for each scenario for matings that were not made and matings that were made; results were similar across replicates. The final birth year was used because it provided the greatest opportunity to generate correlations between inbreeding and number of recessive alleles carried by individuals.

The lack of large correlations between inbreeding and recessive load may be due in part to the low allele frequencies that were used in most scenarios. When deleterious recessives have very low frequencies, the probability that a mating will be affected by more than one recessive locus and that such a mating will have a sufficiently extreme TBV to overcome the penalty, is extremely low. If the frequencies of the recessive alleles were high, a stronger relationship would probably be observed, but a situation in which several recessive alleles are present at a high frequency in the population is extremely unlikely.

Although inbreeding is inevitable in finite populations under selection, e.g., [31], its deleterious effects can be managed if harmful alleles are eliminated from the population. There is evidence that such purging has occurred in cattle populations [32, 33], and in the 100 and 1000 recessive loci scenarios, affected animals were quickly eliminated from the population. As the number of recessive loci in real populations increases, it is more likely that affected matings will occur, resulting in purging that is consistent with the trends in Fig. 6a, b.

### Mate allocation

Mate allocation, i.e., the process of selecting mating pairs from a population of females and some portfolio of males, has a long history in animal breeding programs for both general [16, 18, 34–36] and trait-specific [37] applications. Many AI firms provide mating recommendations to their customers as part of their services, but the algorithms used are usually very simple. Sun et al. [38] recently showed that rates of genetic gain can be increased when genomic relationships and linear programming (LP) are used to assign mates. This may be a more practical way to account for recessive alleles than including them in selection indices because it is difficult to avoid double-counting of costs when several recessives affect fertility early in pregnancy (e.g., double-counting of costs is likely to occur).

An advantage of the modified Pryce method over the original Pryce method is that the former can be used to maintain the frequency of desirable recessive alleles in the population, such as red coat color. There are other recessive alleles, such as slick hair coat [39], that are segregating in some lines of Holstein and are desirable to producers in sub-tropical regions, and the modified Pryce method could be used to increase the frequency of such alleles in the population.

However, both the modified and the original Pryce methods suffer from order-dependence, that is, if the cows are reordered before bulls are allocated, the mate pairs change. This is not a serious problem if the best bulls in the population have similar breeding values, but could become more important if a small group of elite bulls has much higher breeding values than other active bulls. The use of LP could eliminate this problem, at the cost of some added complexity in the implementation phase. Using simulation, Sun et al. [38] found that expected progeny differences were slightly higher when using LP compared to the Pryce method. Progeny inbreeding was also slightly lower using LP. Similar gains using LP to constrain progeny inbreeding were reported by Weigel and Lin [21].

Sequential allocation, as used in the Pryce and modified Pryce algorithms, cannot account for a situation in which the value of one mating is affected by other matings, which is common when matings on multiple farms are considered simultaneously or management of parental coancestry is desired. Van Eenennaam and Kinghorn [15] recently extended the MateSel program [16] to permit selection against the number of lethal alleles and against recessive lethal genotypes. The genetic progress foregone to decrease the incidence of lethal homozygotes depends on allele frequencies, the number of lethal loci, and the emphasis that is placed on avoiding embryonic deaths. Their approach is theoretically more satisfying than the algorithm presented in this paper, but breeding organizations in the US are often reluctant to modify their software. Because of this, ease-of-implementation is often given more importance than theoretically optimal properties, and it is better to have an imperfect mate allocation tool used than no tool at all.

### Integration with on-farm systems

As of 27 July 2015, there were 1,059,438 genotyped individuals in the National Dairy Database maintained by the Council on Dairy Cattle Breeding (Reynoldsburg, OH, USA), of which 854,766 were females (https://www.cdcb.us/Genotype/cur_freq.html). The modified Pryce method described in this paper can be easily integrated into existing herd management and mate planning software, where it can be used in combination with these genotypes to better inform culling decisions or identify matings to be avoided. In the case of some haplotypes, such as A2 beta-casein and polled, this can be a useful tool to increase allele frequencies without sacrificing substantial cumulative genetic gain.

### Trade-offs and limitations

In the modified Pryce scenario, it was possible to reduce but not eliminate embryonic mortality. As the relative weighting (economic value) of loci increases, the amount of genetic progress foregone will also increase. MacArthur et al. [40] recently estimated that each human genome contains approximately 100 loss-of-function mutations and about 20 genes that are completely inactivated. While not all of these mutations are lethal, it suggests that the scenario with 100 loci of Van Eenennaam and Kinghorn [15] represents a plausible upper limit to the selection problem. Segelke et al. [41] suggested that a genetic index that includes haplotypes of interest, either beneficial or harmful, should be used when selecting females for mating, and estimated breeding values for production and fitness traits should be used to select bulls in order to balance selection for specific alleles with genetic gain. As the number of recessive alleles increases, it will become increasingly more difficult to assign proper weights to each of them, and it will be difficult to assign costs to each recessive without double-counting.

## Conclusions

A modified version of the Pryce method [18] that accounts for the economic effects of recessive conditions was developed and compared with random mating, truncation selection, and the Pryce method for several scenarios, including hypothetical recessive lethal loci, as well as 12 recessive loci that are currently segregating in the US Holstein population. The new method appears capable of both reducing the frequency of undesirable recessive alleles with low frequencies and maintaining or increasing the frequency of desirable recessive alleles. The method can be easily implemented in software used for mate allocation, and the code used in this study is freely available for use as a reference implementation.
